# Fever of unknown origin: cost of illness study

**DOI:** 10.1515/med-2026-1409

**Published:** 2026-04-30

**Authors:** Ivana Lesnjak, Biljana Popovska Jovicic, Katarina Jankovic, Aleksandar Canovic, Tijana Stanojkovic, Milan Gajic, Srecko Markovic, Anja Manojlovic, Sara S. Mijailovic, Slobodan M. Jankovic, Ana M. Barjaktarevic, Olivera Z. Kostic, Milovan R. Stevic, Jana V. Mojsilovic, Marina J. Kostic

**Affiliations:** Department of Pharmacology and Toxicology, Faculty of Medical Sciences, University of Kragujevac, Kragujevac, Serbia; Clinic for Infectious Diseases, University Clinical Centre Kragujevac, Kragujevac, Serbia; Department of Infectious Diseases, Faculty of Medical Sciences, University of Kragujevac, Kragujevac, Serbia; Medical Faculty of the Military Medical Academy, University of Defence, Belgrade, Serbia; Department of Medical Statistics and Informatics, Faculty of Medical Sciences, University of Kragujevac, Kragujevac, Serbia; Department of Pharmacy, Faculty of Medical Sciences, University of Kragujevac, Kragujevac, Serbia; Centre for Harm Reduction of Biological and Chemical Hazards, Faculty of Medical Sciences, University of Kragujevac, Kragujevac, Serbia; Department of Dentistry, Faculty of Medical Sciences, University of Kragujevac, Kragujevac, Serbia

**Keywords:** fever of unknown origin, direct costs, cost of illness study, Western Balkans

## Abstract

**Objectives:**

To analyze the structure of direct treatment costs for FUO and identify key cost-driving factors.

**Methods:**

A retrospective pharmacoeconomic analysis using a bottom-up approach was conducted on 71 patients treated for FUO at the Clinic for Infectious Diseases, University Clinical Centre Kragujevac, between 2016 and 2022. Treatment cost data were extracted from the electronic medical records.

**Results:**

The total direct cost was €81,655.20, with a mean of €1,150.07 ± 3,434.60 per patient with a median of €549.55 (IQR=€512.62). Major cost components included pharmacotherapy (49.40 %; €40,337.26), diagnostic procedures (30.52 %; €24,923.44), and hospitalisation (10.32 %; €8,324.00). Significant cost determinants were the following: gender, the Charlson comorbidity index, hospital stay duration, number of medications administered per patient, and number of specialist consultations.

**Conclusions:**

Pharmacotherapy, especially broad-spectrum antibiotics and antifungals, along with diagnostics and hospitalisation, are the main contributors to FUO treatment costs. Patient-specific factors, particularly comorbidities and service utilisation, play a significant role in determining overall healthcare expenditure.

## What is known?

Despite advances in imaging technologies and microbiological diagnostics, the management of patients with fever of unknown origin (FUO) continues to pose significant challenges for healthcare professionals. This complexity often results in increased utilisation of healthcare resources, driven by unnecessary diagnostic investigations, inappropriate empirical treatment, and prolonged hospital admissions, thereby compounding the overall burden associated with the condition.

## What new information does this article contribute?

This study primarily identifies pharmacotherapy, diagnostic procedures, and hospitalisation as the primary cost drivers in the management of fever of unknown origin (FUO) within the health care systems of Western Balkan countries. The findings underscore critical areas for potential cost containment, particularly in resource-constrained healthcare settings.

## Introduction

Fever of unknown origin (FUO) remains a notable diagnostic challenge in clinical practice. It is traditionally defined as a documented temperature of ≥38.3 °C persisting for at least three weeks, without a clear cause despite appropriate diagnostics [1], 2]. Since its initial definition given by Petersdorf and Beeson in 1961, the concept has broadened from a single entity to a set of conditions that can present with unexplained prolonged fever [3], 4]. The current diagnostic criteria for FUO include a documented fever of ≥38.3 °C (101 °F) on at least two occasions, illness duration of three weeks or longer (or recurrent febrile episodes within that time interval), and the exclusion of immunocompromised individuals [1], 5]. More than 200 potential causes of FUO have been identified. These are commonly classified into four categories: infectious diseases, malignancies, non-infectious inflammatory disorders (including autoimmune and rheumatic conditions, vasculitis, and granulomatous diseases), and a miscellaneous group comprising various less frequent causes. Examples from the latter include: subacute thyroiditis, inflammatory bowel disease, drug-related fever, and factitious fever. However, in a subset of patients, despite the thorough evaluation, the underlying cause remains unclear, resulting in cases of truly undiagnosed FUO [6]. In high-income countries, the predominant causes of fever of unknown origin are malignancies and rheumatological disorders, whereas in low- and middle-income countries, infectious diseases continue to represent the most frequent aetiological category [3], 7]. Despite the advances in rapid laboratory diagnostics and the availability of sophisticated imaging technologies, a substantial proportion of fever of unknown origin cases remain undiagnosed. Owing to the broad differential diagnosis, the diagnostic process is often prolonged and involves the extensive and at times indiscriminate use of various investigative procedures. Furthermore, patients with fever of unknown origin typically require extended hospital stays and undergo a wide range of complex and costly diagnostic evaluations, including the following: nuclear magnetic resonance (NMR), computed tomography (CT), ultrasonography (US), radiography, positron emission tomography (PET), as well as numerous serological, microbiological, haematological, and immunological tests [8]. As a result, these investigations generate significant direct and indirect costs associated with the management of fever of unknown origin [9]. Given its prevalence and economic implications – particularly in countries undergoing socioeconomic transition – estimating the direct costs of fever of unknown origin treatment and identifying its key cost drivers could provide valuable insights for healthcare policymakers. Subsequently, such data may assist in prioritising resource allocation for disease management and in formulating targeted prevention strategies.

Given the substantial diagnostic complexity, prolonged hospitalisation, and extensive use of advanced imaging and laboratory investigations associated with FUO, a detailed evaluation of its economic burden is warranted. However, data on the direct costs and key cost determinants of FUO management remain limited, particularly in countries undergoing socioeconomic transition such as the Republic of Serbia. Therefore, we hypothesised that the total direct costs of FUO management were significantly influenced by specific clinical and healthcare resource–related factors, including length of hospital stay, diagnostic procedures, and pharmacological treatment. Accordingly, the aim of this study was to evaluate the total direct costs associated with the management of patients with fever of unknown origin in the central region of the Republic of Serbia and to identify the principal determinants influencing these costs.

## Methods

### Study design

In order to evaluate the pharmacoeconomic aspects of treating fever of unknown origin, a cost-of-illness study employing a bottom-up approach was conducted. This retrospective study was performed from the perspective of the National Health Insurance Fund of the Republic of Serbia. The methodology adhered to the Consolidated Health Economic Evaluation Reporting Standards 2022 (CHEERS 2022) guidelines, as established by the International Society for Pharmacoeconomics and Outcomes Research (ISPOR) [10].

### Study population

Patients hospitalised with the diagnosis code R50 “Fever of other unknown origin” [11] at the Clinic for Infectious Diseases, University Clinical Centre Kragujevac, were included in the study – for the purpose of estimating the total direct costs of treating fever of unknown origin.

**Figure 1:**
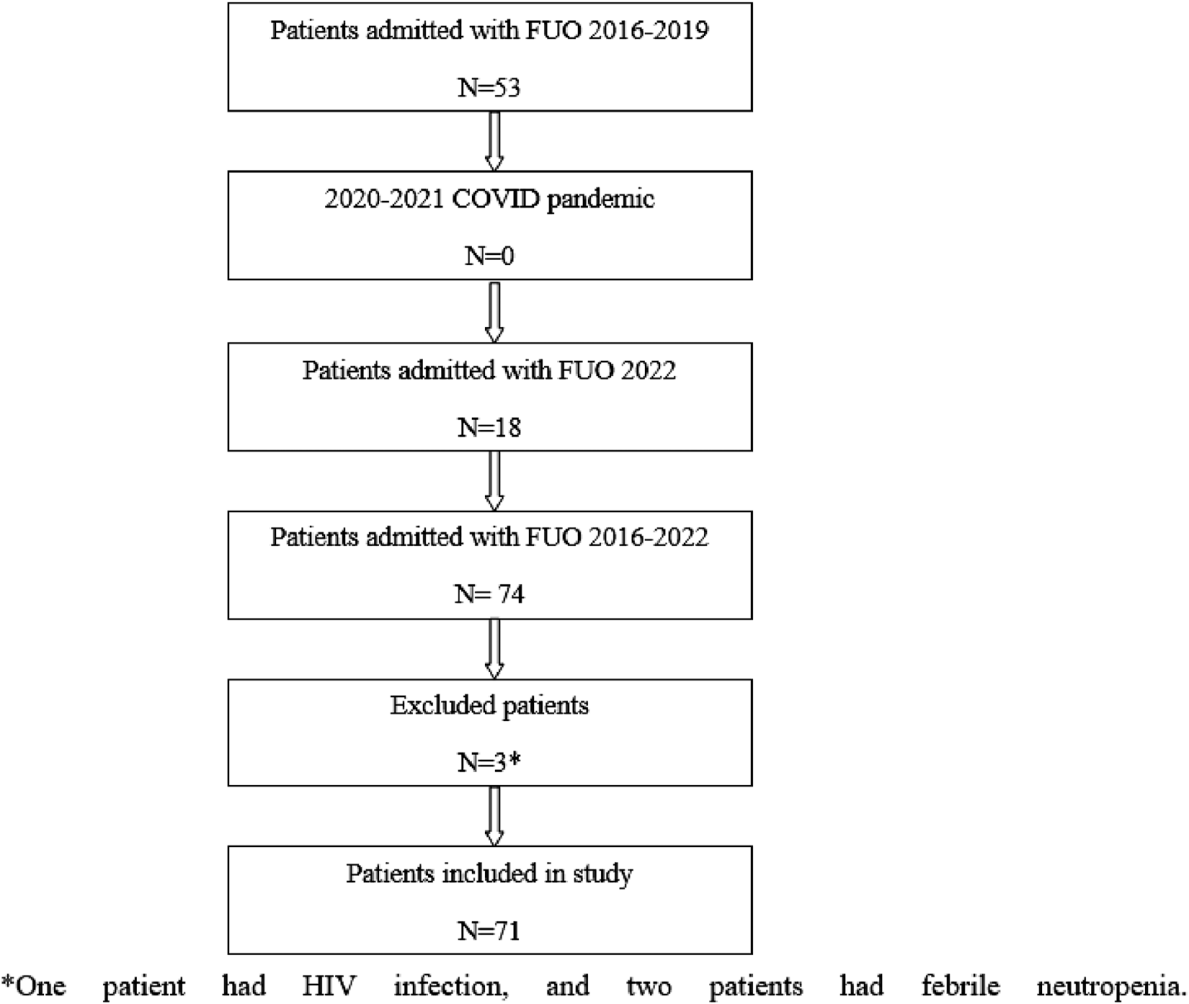
Patient selection flow diagram.

It should be emphasised that no published data provide precise estimates of the total direct costs of FUO to guide sample size calculation. Therefore, we referred to the previously published cost of illness studies of febrile neutropenia [12], as it represents a medical condition with similar diagnostic and therapeutic challenges, prolonged hospitalisations and heterogeneous treatment costs. While these studies cannot directly determine the sample size for FUO, they illustrate the range of variability in costs. Based on these comparisons, our sample of 71 patients was considered sufficient to provide a reasonably precise descriptive estimate of mean costs. All consecutive patients admitted to the University Clinical Centre Kragujevac with FUO between 2016 and 2022, excluding 2020–2021 due to the COVID pandemic, were screened for inclusion. Subsequently, the patients were enrolled in the study if they met all of the predefined inclusion criteria (patients of both sexes with a diagnosis code R50 (*Febris causae ignotae aliae*), complete and accurate medical records, and treatment within the specified period). On the other hand, the patients were excluded if they did not meet the exclusion criteria (patients under 18 years of age, those with HIV infection, malignancies, nosocomial FUO, or febrile neutropenia). A flow diagram illustrates the selection process, including the number of patients who were screened, excluded and finally included in the study (Figure 1).

### Demographic and clinical data

Demographic and clinical information were extracted from the patient medical records. Demographic data included general characteristics such as age and gender, whereas clinical data consisted of the following parameters: duration of fever, length of hospitalisation, the Charlson comorbidity index (CCI) score [13], final discharge diagnosis, treatment outcomes (discharge or continuation), laboratory and imaging investigations, specialist consultations, pharmacotherapy, and hospital care.

### Costs

To evaluate the pharmacoeconomic aspects of FUO treatment, we adopted an institutional perspective, examining all costs from the viewpoint of the National Health Insurance Fund of the Republic of Serbia.

In order to capture the total direct costs of fever of unknown origin treatment, medical data concerning healthcare service utilisation, consumables, and pharmaceuticals were translated into pharmacoeconomic data using the Tariff Book and drug pricing information from the electronic records provided by the Republic Fund of Health Insurance websites [14], 15]. In addition, treatment costs were initially recorded in Serbian dinars (RSD) and subsequently converted to euros (€) using the average exchange rate (December 2022th), published by the National Bank of Serbia.

### Statistical analysis

All data extracted from the patients’ medical records were analysed using IBM SPSS Statistics for Windows, version 21.0 (IBM Corp., Armonk, NY, USA) [16]. Additionally, descriptive statistics, including measures of central tendency (mean, median) and dispersion (standard deviation, minimum, maximum, and interquartile range), were calculated to summarise the data. Furthermore, the distribution of continuous variables was assessed using the Kolmogorov–Smirnov test. As the data were not normally distributed, the Mann–Whitney U test was used for comparisons between two groups. Subsequently, in order to determine the influence of individual determinants on the total treatment costs, a generalised linear model was implemented, including the following variables in the model: sex, discharge diagnosis, age, Charlson comorbidity index (CCI) score, length of hospitalisation, number of drug units per patient, number of infectious disease examinations, and number of consulting examinations.

### Ethical approval

Ethical approval was granted by the Ethics Committee of the Clinical Centre Kragujevac (Authorization No. 01/18-843).

## Results

This study enrolled 71 patients presenting with fever of unknown origin (FUO), comprising 42 women (59.2 %) and 29 men (40.8 %), with a mean age of 49.4 years (range, 24–85 years). Table 1 summarises the patients’ clinical characteristics, including the final discharge diagnosis, frequency and percentage of discharge vs. the continuation of therapy, duration of fever, length of hospitalisation, and presence of comorbidities expressed as the Charlson comorbidity index score/percent.

**Table 1:** Clinical characteristics of patients with FUO.

Clinical characteristics	n (%)
Discharge diagnosis	
Infectious	23 (32.4 %)
Non-infectious	40 (56.3 %)
Other	8 (11.3 %)
Hospital discharge disposition	
Discharge	64 (90.1 %)
Continuation of treatment	7 (9.9 %)

**Clinical characteristics**	**Mean ± SD**

Length of hospitalisation (number of days)	13.24 ± 10.75
Duration of fever (number of days)	73.32 ± 84.47
Charlson index score	1.06 ± 1.42
Charlson index percent	90.11 ± 17.59

**Figure 2:**
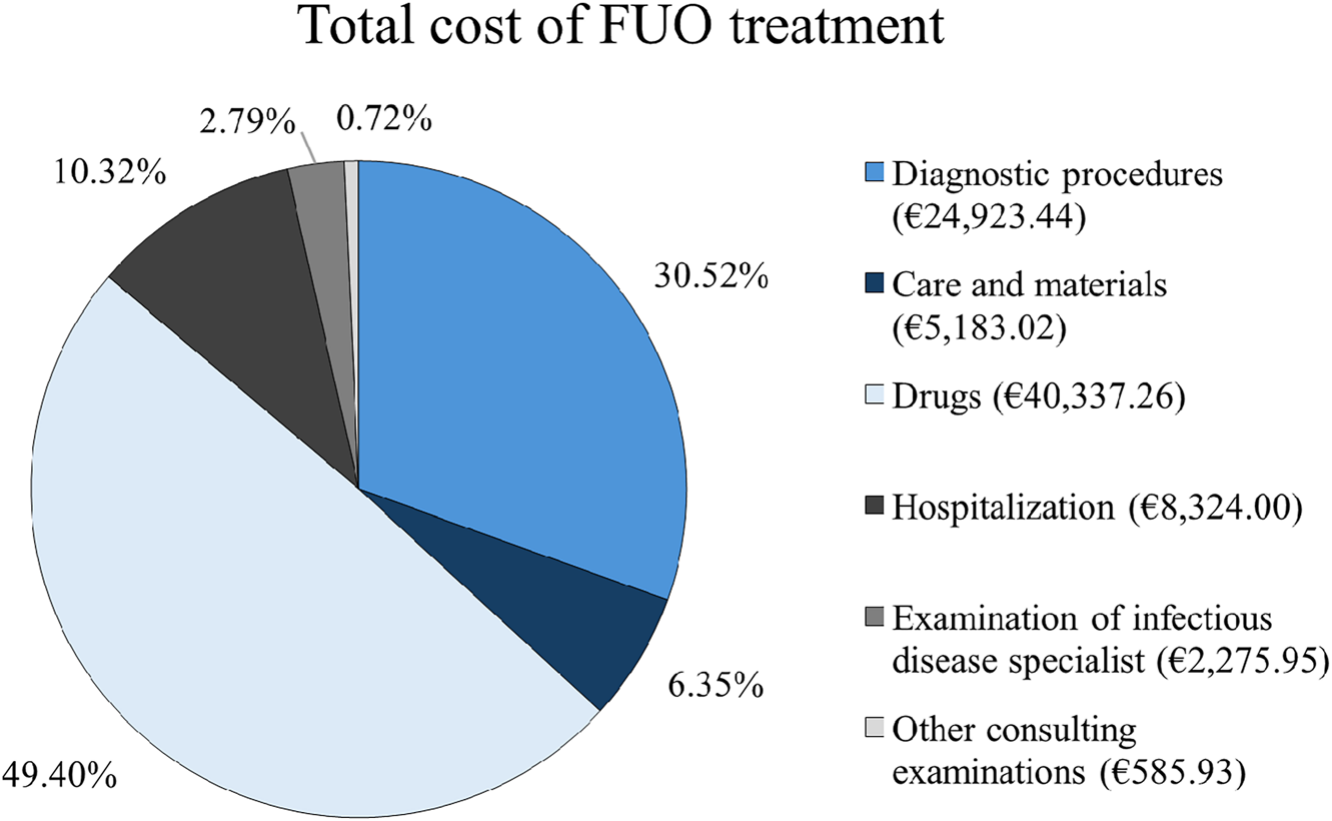
Structure of the total cost of FUO treatment.

The total direct costs of treating FUO for all participants in our study were estimated at €81,655.20, while the mean costs of treating FUO per patient was €1,150.07 ± 3,434.60 with a median of €549.55 (IQR=€512.62). The largest determinants among direct costs of treating FUO were related to the costs of pharmacotherapy, diagnostic procedures and hospitalisations with 49.40 %, 30.52 % and 10.32 % respectively (€40,337.26, €24,923.44 and €8,324.00). Additional cost components included caregiving and materials (6.35 %, €5,183.02), – infectious disease specialist consultations (2.79 %, €2,275.95), and specialist consultations (0.72 %, €585.93) (Figure 2). Descriptive statistics – including the median, minimum, and maximum values – for each of the cost determinants are specified in Table 2.

**Table 2:** Median, minimum, and maximum values of determinants of the total treatment costs among patients with FUO.

Total costs of the main determinants of treatment of FUO	Median (IQR) (€)	Min (€)	Max (€)
Diagnostic procedures	262.37 (254.47)	71.22	2,724.43
Drugs	57.20 (123.97)	0.006	28,014.41
Hospitalisation	117.59 (139.57)	1.86	991.60
Care and materials	28.13 (57.59)	0.79	749.98
Examination of infectious disease specialist	24.21 (21.75)	7.26	205.80
Other consulting examinations	7.26 (9.68)	2.42	33.89

Regarding the total costs of diagnostic procedures, the results of this study reveals that the largest proportions were attributable to complete blood counts with biochemical analyses (24.43 %) and diagnostic radiology (23.72 %) (€6,088.08 and €5,911.32, respectively). Cumulatively, other pharmacoeconomic determinants related to diagnostic procedures were presented in the total direct costs with 11.37 % (€2,834.40) (bacteriological analysis), then 10.04 % (€2,503.19) (diagnostic virology), 7.89 % (€1,967.42) (immunology diagnostic tests), 5.6 % (€1,395.22) (invasive diagnostic procedures), 5.51 % (€1,373.95) (coagulation tests), 4.27 % (€1,063.28) (tumour marker tests), 3.24 % (€808.20) (serology tests), 2.28 % (€567.36) (nuclear medicine tests) and 1.65 % (€410.97) (other diagnostic procedures).

Diagnostic radiology was predominantly driven by ultrasound studies (59.41 %, €3,512.17), followed by computed tomography (20.53 %, €1,213.64) and plain radiography (12.86 %, €760.16), with magnetic resonance imaging (MRI) and other diagnostics constituting the lowest share (3.6 %, €212.54). Detailed descriptive statistics – including the median, minimum, and maximum values for each diagnostic modality and the total number of procedures are presented in Table 3.

**Table 3:** Median, minimum, and maximum values of costs and number of diagnostic procedures by type of radiological examinations.

Types of diagnostics	n of diagnostic procedures	% of total diagnostic costs	Mean number of procedures per patient	Median (IQR) (€)	Min (€)	Max (€)
Diagnostic radiology						
X-ray	87	3.05	1.23	8.60	8.01	60.23
US	165	14.09	2.32	39.38	0.82	116.76
CT	43	4.87	0.61	30.37	16.41	121.44
NMR	5	0.85	0.07	21.75	17.06	134.90
Other diagnostic radiology types	8	0.85	0.11	29.83	29.83	33.75
Invasive diagnostic procedures	49	5.60	0.69	48.8	12.27	198.63
Bacteriological analysis	383	11.37	5.39	17.16	1.34	925.67
Diagnostic virology	75	10.04	1.06	37.60	5.95	153.82
Serology tests	242	3.24	3.41	17.76	6.19	93.78
Immunology diagnostic tests	445	7.89	6.27	34.26	6.65	72.79
Tumour marker tests	181	4.27	2.55	23.01	6.15	48.99
Blood cell counts and blood chemistry panels	2,906	24.43	40.93	54.34	9.13	923.86
Coagulation tests	311	5.51	4.38	21.28	1.87	231.87
Nuclear medicine tests	132	2.28	1.86	7.86	4.08	60.17
Other diagnostic procedures	38	1.65	0.54	4.78	2.18	201.02
Total	5,070	100.00	71.41	262.37	71.22	2,724.43

IQR, the interquartile range.

Antifungal therapy constituted the majority of total drug-related expenditures, accounting for 65.5 % (€26,440.87), followed by antibiotics (15.1 %, €6,099.62), anticoagulants (3.4 %, €1,379.78), gastroprotective agents (2.5 %, €993.71), nonsteroidal anti-inflammatory drugs and antipyretics (0.8 %, €321.71), vitamins (0.5 %, €207.41), antivirals (0.1 %, €29.98), and all other medications combined (0.05 %, €19.91). Table 4 presents the total number of used drug units, with corresponding median, minimum and maximum costs.

**Table 4:** Total number of used drug units and costs.

Groups of drugs	Number of drug units	Mean number of drugs per patient	Median (IQR) (€)	Min (€)	Max (€)
NSAIL and antipyretics	629	8.86	3.63 (5.91)	0.06	67.41
Antibiotics	1,990	28.02	47.02 (187.76)	2.13	1,249.18
Antifungals	391	5.51	26.57 (47.43)	1.60	26,214.23
Antivirotics	10	0.14	–	–	–
Gastro-protectives	690	9.72	22.34 (40.00)	0.068	138.30
Anticoagulants	337	4.75	55 (87.12)	0.74	727.31
Vitamins	339	4.77	3.82 (7.65)	0.63	88.12
Corticosteroids	75	1.06	1.73 (6.81)	0.07	9.70
Antihypertensives and antiarrhythmics	559	7.87	1.87 (7.50)	0.03	43.53
Sedatives	204	2.88	1.23 (2.21)	0.04	4.93
Supportive therapy	4,256	59.94	47.24 (295.70)	0.04	914.15
Other drugs	996	14.03	1.44 (8.97)	0.13	34.69
Total	10,475	147.53	57.20 (123.97)	0.06	28,014.41

IQR, the interquartile range.

Regarding the types of FUO, the infectious type accounted for the highest share of total treatment costs, representing 55.91 % (€45,651.42), followed by the non-infectious type with 38.25 % (€31,229.62). Other diagnoses contributed 5.85 % (€4,774.15) of the total cost. No statistically significant differences in the main determinants of total treatment costs for FUO were observed according to the discharge diagnosis (the Kruskal–Wallis test, p>0.05), as specified in Table 5.

**Table 5:** Comparison of treatment costs among the types of FUO.

Total costs of the main determinants	Infective median (IQR) (€)	Non-infective median (IQR) (€)	Other median (IQR) (€)
Diagnostics	302.17 (298.01)	278.45 (196.84)	232.99 (251.86)
Drugs	65.38 (389.75)	59.73 (101.29)	43.37 (102.60)
Hospitalisation	55.72 (290.07)	28.99 (137.47)	49.01 (119.85)
Care and materials	22.05 (93.56)	28.40 (48.43)	17.57 (88.23)
Infectious disease specialist examinations	24.21 (29.05)	26.63 (21.79)	20.58 (2.42)
Consulting examinations	7.26 (8.47)	7.26 (7.26)	6.05 (14.53)
Total	597.93 (1,013.66)	517.73 (404.08)	500.84 (515.63)

IQR, the interquartile range.

**Figure 3:**
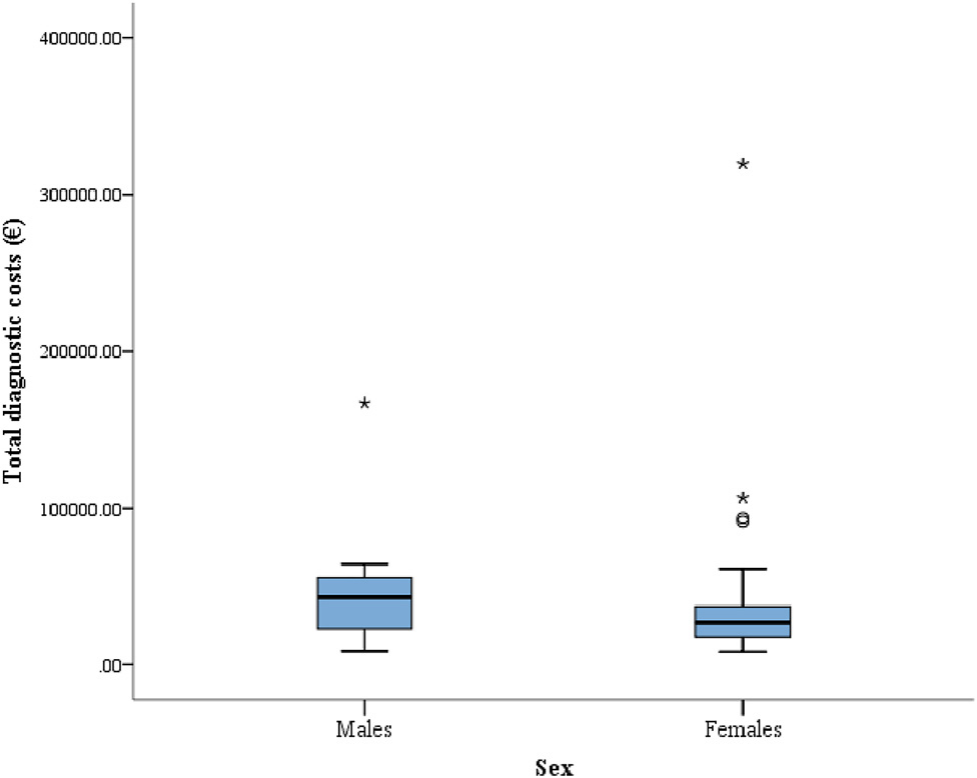
Distribution of the total diagnostic costs of treating FUO by sex.

A significant positive correlation was found between the total treatment costs and both length of hospitalisation (ro=0.539; p<0.001) and the Charlson comorbidity index score (ro=0.324; p<0.05), whereas age and duration of fever were not significantly associated with the total treatment costs (p>0.05). Furthermore, cost comparison analyses between female and male patients revealed statistically significant differences in diagnostic expenses, care and materials, and the overall treatment costs (p<0.05). Specifically, male patients had significantly higher diagnostic costs (a median of €368.29 vs. €227.63) (Figure 3), care and material expenses (a median of €40.12 vs. €18.27), and the total treatment costs (a median of €644.12 vs. €435.16) compared to female patients.

To determine the influence of individual determinants on the total treatment costs, a generalised linear model was implemented, including the following variables: sex, discharge diagnosis, age, the Charlson comorbidity index score, length of hospitalisation, number of drug units per patient, number of infectious disease specialist examinations, and number of consulting examinations. The analysis adequately describes the influence of the studied variables on the total treatment costs (Deviance=15.205; Pearson Chi-square=17.601; AIC=1,701.48) and the model is statistically significant (p<0.001). Significant factors identified in the model were as follows: sex, the Charlson comorbidity index score, length of hospitalisation, number of drug units per patient, and number of consulting examinations and their influence on the total costs of treating FUO, as shown in Table 6.

**Table 6:** Influence of determinants on the total treatment costs.

Characteristics	B	95 % CI	Wald Chi-square
Sex^a^	0.334	9.640–10.916	7.029
Discharge diagnosis	0.322	−0.066–0.709	2.647
Age	−0.005	−0.018–0.008	0.610
Charlson index score^a^	0.182	0.036–0.329	5.962
Length of hospitalisation^a^	0.018	0.004–0.031	6.463
Duration of fever	0.001	0.000–0.003	2.208
Number of drug units^b^	0.002	0.001–0.002	42.664
Number of consulting examinations^a^	0.065	0.017–0.114	6.891

CI, a confidence interval; ^a^p<0.05; ^b^p<0.001.

## Discussion

In the present study, we found that the management of fever of unknown origin is associated with substantial direct healthcare costs, primarily driven by pharmacotherapy, particularly broad-spectrum antibiotic use along with diagnostic procedures and prolonged hospitalisation. Furthermore, patient-specific factors, especially the presence of comorbidities and the intensity of healthcare service utilisation, were significant determinants of the overall treatment costs. These findings underscore the considerable economic burden of FUO within the socioeconomic context of a country in the Western Balkans. Given the diverse aetiologies and complex clinical presentation, fever of unknown origin poses a significant challenge to clinicians, not only in terms of diagnosis and management, but also from a health economic standpoint [17]. Evaluating FUO from a pharmacoeconomic perspective may help identify modifiable cost drivers, the optimisation of which could contribute to more efficient and cost-effective management strategies.

The study found that the mean cost of FUO treatment in Serbia was €1,150.07 ± 3,434.60 per patient. When compared with the available literature, this figure varies: the costs in Sri Lanka (Premathilaka et al.) were lower (€407.08), whereas those in Spain and Australia were significantly higher – approximately 9.7 and 4.2 times greater, respectively (€11,167 and €4,795 per patient). In contrast, the findings of Chen et al. closely align with those observed in our study. These discrepancies may reflect differing healthcare service valuations across various systems [9], [18], [19], [20]. It is noteworthy that while pharmaceutical prices remain relatively consistent with those in the Serbian healthcare system, valuations of health services vary considerably across EU countries. This disparity explains the differences observed in FUO diagnostic and treatment costs. For example, a hospital day in Serbia typically ranges between €10 and €35, which is significantly lower than the EU average [17], 21].

Differences in the costs per patient can largely be attributed to how the total costs were structured and evaluated, in addition to using different methods of monetary valuation. For instance, Premathilaka et al. identified diagnostic procedures as the main cost driver in FUO management [9]. However, pharmacotherapy was the dominant contributor in our study. Similarly, Becerra Nakayo et al. reported comparable diagnostic cost patterns, in line with our findings [18]. In our cohort, pharmacotherapy, diagnostics, and hospitalisation accounted for 49.40 %, 30.52 %, and 10.32 % of direct treatment costs, respectively. This distribution reflects the complex clinical nature of FUO, which often involves prolonged diagnostic investigations over several weeks or months [8], 22]. Previous studies have highlighted the cost-effectiveness of FDG-PET/CT in the FUO diagnostic process, due to its ability to detect underlying causes with greater speed and accuracy. This reduces the need for additional expensive tests and shortens hospital stays, thereby lowering total treatment costs. Nonetheless, its use presupposes access to imaging infrastructure and appropriate clinical protocols – factors that depend on a healthcare system’s economic and organisational capacity [17], [18], [19], [20, 23].

Regarding pharmacotherapy, the largest share of costs in our cohort was associated with antifungal and antibiotic agents. The unclear aetiology and non-specific clinical/laboratory features of FUO often necessitate empirical use of broad-spectrum antimicrobials. However, the high antifungal costs in our study were largely driven by one male patient with classic FUO due to cryptococcal meningitis, who required prolonged hospitalization and intensive antifungal therapy. The higher costs observed in male patients were primarily influenced by this exceptional case and the intensity of treatment required, rather than a general sex-based difference or comorbidities. These findings illustrate how rare but high-cost cases can significantly impact pharmacoeconomic analyses in FUO, reflecting variability in resource utilisation in real-world clinical practice. The published literature also supports the role of empirical antifungal treatment in febrile neutropenic patients, especially when antibiotic therapy proves ineffective [24].

Key factors influencing the total treatment costs in our study included the following: gender, the Charlson comorbidity index score, length of hospitalisation, number of medications per patient, and frequency of specialist consultations.

Although the cohort included predominantly non-elderly patients, we used the CCI as a standardized measure of co-morbidity burden, reflecting clinical complexity and its association with increased healthcare utilisation. Higher CCI scores were linked to greater resource use, including more frequent specialist consultations, more prescribed medications, and higher total costs. These findings suggest that the CCI can help identify FUO patients at higher risk of prolonged hospitalisation or intensive interventions, supporting its potential role in risk stratification and resource allocation. Previous research also supports its utility in prospectively identifying patients likely to incur higher treatment costs [25]. Our findings further highlight the economic impact of repeated specialist consultations, which are essential for monitoring treatment progress and adjusting therapy. Complex FUO cases often require multiple consultations, resulting in increased resource use. As anticipated, the greater involvement of physicians in diagnostic and treatment processes was associated with higher service use and more prescribed medications, both of which correlated with the increased total costs [26].

## Conclusions

This study highlighted that hospitalisations, diagnostic procedures, and broad-spectrum antimicrobial drugs were the main contributors to the direct costs of treating FUO. Patient characteristics, such as comorbidities and the number of specialist consultations, also significantly influenced the total healthcare expenditure. Understanding these costs can guide resource allocation strategies, including tailored diagnostic and therapeutic approaches as well. Ultimately, it can improve antimicrobial stewardship, and streamline care pathways to enhance the cost-effectiveness of managing FUO within the healthcare context of the Western Balkans which have a recent history of socioeconomic transition. For future research, prospective studies and cost-effectiveness analyses focusing on patients with higher co-morbidity burdens would be valuable to inform both clinical practice and policy decision-making in FUO management.
